# Socio-economic factors associated with mental health disorders in Fort Portal, western Uganda

**DOI:** 10.4102/sajpsychiatry.v26i0.1391

**Published:** 2020-07-07

**Authors:** Charlotte Hawkins, John M. Bwanika, Martin Ibanda

**Affiliations:** 1 Department of Anthropology, University College London, London, United Kingdom; 2 The Medical Concierge Group Limited, Kampala, Uganda; 3 Mental Health Unit, Fort Portal Regional Referral Hospital, Fort Portal, Uganda

**Keywords:** mental health, mental health services, stigma, economic stress, outreach, Uganda

## Abstract

**Background:**

Mental health disorders, which are interlinked with social issues such as poverty and stigma, present a significant burden in Uganda.

**Aim:**

This article explores perceptions about and experiences of mental health disorders in western Uganda, particularly as they pertain to the socio-economic context.

**Setting:**

The research was conducted in the mental health unit at the Fort Portal Regional Referral Hospital, Kabarole District, Uganda.

**Method:**

This article is based on qualitative anthropological research conducted from January to March 2017, including 49 semi-structured interviews about ideas and determinants of mental health, with health workers, former mental health service users, their relatives and influential community members.

**Results:**

Many interviewees felt that mental health disorders are an increasing problem in their community. Economic challenges, such as poverty, unemployment and financial stress, are seen as both a cause and a consequence of mental illness. Mental health challenges can be exacerbated by shortages in mental healthcare, which are shown to be complexly interrelated with stigma.

**Conclusion:**

This article provides an insight into mental health experiences in Fort Portal based on the perspectives of various interviewees. Further funding and research are recommended to inform contextually appropriate services.

## Introduction

According to the 2010 global burden of disease survey, mental, neurological and substance use (MNS) disorders are leading causes of the disease burden, responsible for 10.4% of ‘Disability-Adjusted Life Years’ (DALYs).^1^ The World Health Organization (WHO) recognises an ‘escalating burden of mental disorders’ around the world, which are determined by both individual attributes and broader contextual issues.^2^

Socio-economic factors are particularly influential in determining both mental health disorders and the effectiveness of mental health policies and services. The mental health burden is therefore disproportionately felt in low-income countries, where poverty is prevalent, and psychiatric specialists, medicines and responsive community-based services are lacking, as stated by the WHO.^3^ Accessible and cost-effective mental health interventions need to respond to an evidence base of contextual considerations.

Whilst there have been no systematic reviews on the prevalence of mental health problems in Uganda to date,^4^ and there is limited reliable data on hospital utilisation,^5^ the WHO has estimated that there are a total of 2.2 million people affected by MNS disorders in Uganda, with only 9% of them able to access care.^6^ According to 2017 data, there are 0.08 psychiatrists for every 100 000 people, as well as 2.24 mental health nurses and 0.5 ‘other paid mental health workers’.^7^ In the Kabarole district where this study was based, a 2002 survey of 384 households identified 130 individuals with a mental health disorder in the previous year, leading them to estimate that 31% of the population experience mental health disorders.^8^ Whilst the sample is limited, it suggests that mental health problems are endemic in the district.

Uganda has a high population growth of 3.2%, with a 2018 IndexMundi estimate showing that 69% of the population is under the age of 25.^9^ Uganda is classed by the World Bank as a low-income country,^10^ and their assessment notes that whilst poverty rates have decreased from 57% in 1993 to 20% in 2013, inequality is rising and ‘even after two decades of progress, poverty is still widespread’.^11^ Epidemiology often determines that poverty correlates with higher rates of disease,^12^ and WHO cites various studies that confirm this relationship between poverty and mental illness.^13^ A 2019 study in six low- and middle-income countries, including Uganda, found that households with a member with an MNS disorder had lower income and higher healthcare expenditure, leading the researchers to conclude that households that have a member with an MNS disorder are ‘susceptible to chronic poverty’.^14^ Psychologists have defined poverty-induced breakdown as chronic stress or ‘toxic shock’, a response to ‘strong, frequent, and/or prolonged adversity’,^15^ which damages ‘brain architecture’ and future health. Furthermore, theorists note that mental illness and poverty also often exist in a cyclical relationship with stigma,^16^ notably so in Uganda.^17^ This suggests that systemic issues can impact individuals’ mental health, supporting the relevance of the current study, which is an exploration of the experiences of mental health disorders in relation to the social and economic context in Fort Portal, from the perspective of health workers, former service users and their relatives.

## Research methods and design

This qualitative study used face-to-face, informal, in-depth interviews to collect responses from 49 participants chosen on a judgement basis, or selecting respondents most appropriate to the research interests.^18^

Health workers, mental health service users and their relatives were interviewed by the principal investigator for their views and experiences of mental health problems in the context of the Kabarole District. Service users interviewed were formerly in-patients at the hospital and act as peer support workers to assist the hospital with community-based care. Given the subjectivity and complexity of research interests, holistic qualitative methods such as open-ended interview questions were particularly relevant.^19^ Research design and implementation was supported by the psychiatric clinical officer (PCO) in-charge at the hospital, who facilitated the research in the mental health unit and assisted with the recruitment of interviewees. A research assistant who works in the mental health unit as an occupational therapist supported data collection with interview question design and translation into Rutooro. An analysis of the diverse perspectives of respondents can inform an understanding of the socio-economic determinants of mental health problems in Uganda and contribute to the development of contextually relevant mental health interventions, such as social psychiatry and public health progammes.^20^

### Setting

**FIGURE 1:**
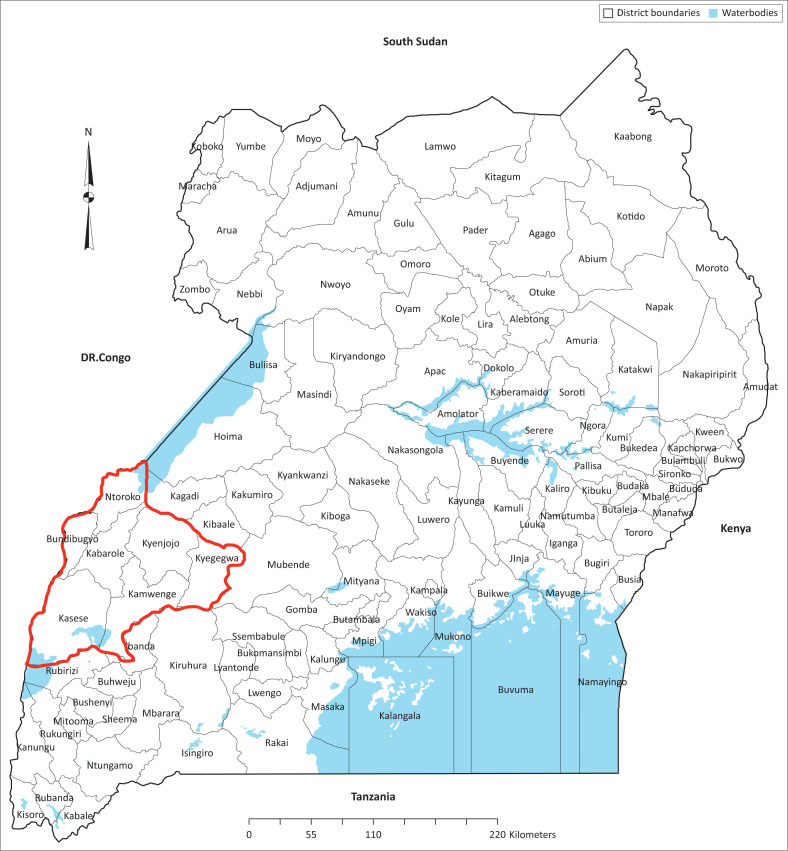
Map from Uganda Bureau of Statistics 2014 Census, relevant districts outlined in red.^21^

This research was conducted in the psychiatric unit within a general regional hospital in the town of Fort Portal, Kabarole district, western Uganda. The hospital serves the seven surrounding districts, including part of eastern Democratic Republic of Congo (DRC), comprising 2.2 million people over an area of 11 000 km^2^ (Figure 1). Patients are referred to the regional hospital from all health service levels, including general hospitals, primary healthcare centres and village health teams.^1^ The mental health unit has a bed capacity of 45 in-patients and a busy outpatient clinic, which at the time of the study in 2017 was frequented by approximately 930 people seeking treatment each month. As it was founded in 2005, the staff strength, led by the PCO in-charge, has increased from 2 to 24, and patient intake continues to increase.

### Study population and sampling

Interviewees were recruited using a judgement sampling approach, selecting respondents most appropriate to the research interests,^18^ based on the experience and the network of the PCO in-charge and the research assistant, an occupational therapist. This included their colleagues at the hospital, service users or peer support workers, existing patient’s attendant relatives and key influential members of the community, including religious leaders. Chosen sample categories were additionally informed by Weiss’ framework for researching health concepts and interventions.^22^ This included their colleagues, service users or peer support workers, existing patient’s attendant relatives and key influential members of the community, including religious leaders. Interviewees by population, age and gender are outlined in Table 1.

**TABLE 1:** Interviewees by population, age and gender.

Sample categories	Total	Female			Male		
		20–34	35–49	50+	20–34	35–49	50+
Psychiatric clinical officer	3	-	1	-	-	-	2
Mental health nurse	4	1	3	-	-	-	-
Occupational therapist	2	1	1	-	-	-	-
General health workers (doctors, nurses, students)	5	1	2	1	-	1	-
Hospital social worker	1	-	1	-	-	-	-
Hospital administration	2	-	-	1	-	1	-
Private sector health worker	1	-	-	-	-	1	-
Non-governmental sector health worker	2	-	-	-	1	1	-
Traditional healers	2	-	-	-	-	-	2
Attendant relatives	6	-	2	-	-	3	1
Former service users	12	3	4	-	1	4	-
Community members (e.g. market vendors, boda drivers)	5	-	2	1	-	2	-
Local leaders (e.g. government officials)	2	-	-	-	1	-	1
Religious leaders	2	-	-	-	-	-	2

### Data collection

In-depth, semi-structured interviews lasting 1 h on average were conducted in an informal manner to allow for participants’ interpretation of the questions and to elucidate free opinions.^18^ Interviews mostly took place in an office in the mental health unit. Questions were developed after 2 weeks of participant observation in the mental health unit and adapted with feedback from hospital workers. They explored the following areas of particular relevance in this setting: stories and experiences of mental health disorders; perceptions of causality; treatment and health education preferences; attitudes towards medicine; family and community responses; stigma, exclusion and labelling; self-perceptions; the mental health system in the region; influence of communicable diseases such as human immunodeficiency virus (HIV); and perceptions of neurological disorders such as epilepsy.

### Data analysis

Interviews were recorded using detailed field notes and audio recordings that were transcribed verbatim. Data content was analysed and coded manually by the principal researcher. Data were aggregated into themes that emerged during the coding process, according to Tesch’s procedure.^23^ Validity was ensured through triangulation of findings from the varied interviews and participant observation.^18^ Findings were checked by the PCO in-charge to confirm the accuracy of reporting.

### Ethical consideration

This study protocol was granted full approval by the Makerere University School of Social Sciences Research Ethics Committee (MAKSS REC) on 19 January 2017 (SS71ES). Research activities at Fort Portal Regional Referral Hospital were approved by the hospital director. All research participants gave informed consent to be involved in this study. Pseudonyms have been used or names omitted to preserve confidentiality.

## Results

Many interviewees felt that their socio-economic setting was responsible for a rise in mental health disorders, and the experience worsened by the meanings attached to it. The views of interviewees will be grouped into (1) perspectives on socio-economic causes of mental health disorders, (2) economic consequences of mental health disorders and (3) issues relating to mental healthcare services.

### Socio-economic causes of mental health problems

Many interviewees traced mental health disorders to the restricted ability to personally mediate conditions of economic adversity. Thirty per cent (*n* = 14) cited stress, disappointment and boredom resulting from the economy as the primary cause of mental illness in Uganda, as, for example, in this emphatic description of economic stress in rural areas, stated by the project’s research assistant, an experienced female occupational therapist and mental health worker:

‘The socio-economic disturbance. Initially people in the villages used to not buy food. But now because of climate change, seasons, people buy food, right from the village. That is the stress. They don’t have food for the family the children are crying for it, it is a stress. So you stay stressed. Diseases. You don’t have money. You come to a so-called free hospital there are no drugs. You’re stressed. You’re sick in severe depression, then the coming of HIV, she has four children they all die, they leave young children for her she has no income to buy for them, she’s very old. That is another stressor.’

Here, she refers to the contextual factors outlined above that contribute to the ‘socio-economic disturbances’ around mental health in Uganda. The catalogue of ‘stressors’ and the iteration of the word ‘stress’ reflect the insistent reality the word represents, pressure imposed by unmanageable financial hardship. It is therefore telling that the word ‘stress’ occurs 52 times across transcribed interview data.

Implicit and explicit references to stress were often (*n* = 8) accompanied by expressions of ‘too many’ or ‘too few’ thoughts, as in this citation from a female mental health nurse in her 30s:

‘These days are stressed, people don’t have money. These days disappointments… These days HIV is too much. People they don’t have thoughts, they don’t have everything so stress again.’

Or from this young female former mental health service user and peer support worker…:

‘Maybe people are getting depressed because of modern problems … poverty, not everyone has his own thoughts. They may think of something and it disturbs him, he has no solution, then he gets mad.’

Mental health disorders are also attributed to ‘thinking too much’ about adversity. A Congolese attendant relative described the cause of his brother’s psychiatric problems, and the reason that he had brought him to the mental health unit in Fort Portal; ‘[h]e has seen too much and thought too much about it. There are many events to deal with every day’.

Underemployment was another socio-economic issue (*n* = 10) commonly attributed to mental health problems, sometimes recognised as a contributing factor to the use of drugs and alcohol (*n* = 8), as with the older female hospital administrator here, who lamented, ‘that idleness, that lack of what to do, so people have resorted to take drugs’, or a physical health worker in her 40s who remarked, ‘most of them they get so many certificates. Now you find a big number of the youth who are getting into this drug abuse, this alcoholism’.

### Economic consequences of mental health disorders

*Mulalu* is the word used to refer to people with mental health disorders in Uganda, which can literally translate as ‘mad’, ‘crazy’ or ‘insane’.^i^ People decreed *mulalu* can struggle to get married or gain access to employment and education, thereby preventing them from living what is deemed a progressive and productive life. As a female mental health nurse explained, ‘[*t*]hey say, “that one cannot, that one is mulalu….” But if already you’re sick and someone calls you that name, it can cause you a relapse’.

Another older female mental health worker described this as ‘their double disability’. There were various examples of these ‘double disabilities’ (*n* = 20), the exclusion and illness resulting from the imposed label of *mulalu*. A former in-patient in the mental health unit discussed her challenges in getting a job, the stigma she faces from her family as a result, and the impact this has on her mental health:

‘In my family there’s some stigma, they know I’m not getting a job, so they under look me. Another thing they start nicknaming me, saying such things. They can say oh “mulalu,” they’re naming you that you’re mad, they can start talking bad words on you…When people know you’re mentally sick, they won’t give you a job, you have to hide it. Sometimes I get challenges, when they hurt me, when they disconnect me, I can think a lot of thoughts, then I can ask myself that “eh might I relapse?”’

Similarly, a young male service user had challenges within his community, and struggled to find a job and a wife, despite the fact that his epilepsy and schizophrenia had stabilised because of medication. As he said:

‘They can work, but the jobs are not easy to get and people are stigmatising them… the community is not good for me. Because they always are passing me in the road, when you make a mistake “that one is a mad person,” you overhear them talking about you when you are passing… the problem with those people in the village, the moment they know that you have stepped in a hospital for “mad people,” they know that is a case, you are a “mulalu”…They wouldn’t even think I could spend an hour working.’

In the meantime, he is financially reliant on his mother, which shows how mental health problems can also have economic consequences for caregivers. The mental health unit relies on attendant relatives to provide patients in the hospital with food, bedding and day-to-day care, which means they need to sacrifice working hours and income to take care of the patient. The hospital social worker, a woman in her 30s, is responsible for patients without family support. She explained the consequence of family rejection for patients:

‘In some cases there are patients without family. They reject them and leave them half way to the hospital. There is very little support available from the state in these cases.’

Families with a member living with a mental health disorder can also be labelled and stigmatised. For example, an occupational therapist explained that the relatives of someone who has committed suicide becomes labelled as ‘the suicidal family’; ‘they have to change the children’s names’.

### Challenges in mental healthcare

#### Stigma

Health workers interviewed in the mental health unit at Fort Portal Hospital (*n* = 9) felt that the needs of people with mental health disorders are overlooked by institutional decision-makers, meaning that funding for mental healthcare is neglected. As the female occupational therapist in the mental health unit said, ‘the institution has stigma at all levels, at community levels, at national, it has stigma’. Some mental health workers were frustrated by resource shortages resulting from external decisions and felt that their work ‘doesn’t attract attention because it doesn’t kill like HIV’.

Furthermore, mental health workers explained that they were stigmatised owing to their proximity to people with mental illness, and that their work was accorded a ‘low status in the community’. A senior male PCO at the mental health unit said, ‘they think we are mental like our patients’, which was reaffirmed by a private sector health worker, a man in his 40s:

‘…[*T*]hey tend to think people who work with them are mad. It’s sort of kind of an attitude that people have that mental health is for mad people. The workers they’re there, and the patients, the same, so that’s why they probably don’t give it the respect that it deserves.’

This has implications for attracting much needed medical staff into the field. One young male trainee doctor explained that he would not choose to work in psychiatry, because:

‘…[*I*]n real life in Africa, most of the people tend towards generating income, and then generating income in psychiatry is not so big as compared to other practices.’

Many (*n* = 14) health workers were proponents of health outreach and sensitisation to educate communities about mental health, to destigmatise mental health disorders and to introduce available services at the hospital. As a female HIV nurse explained:

‘…[*I*]f they see a medical personnel then they say, “so you mean it is not contagious?” We need to go and see their reaction, they can say “pardon, explain further”.’

Whilst the funding for community education was not available, mental health workers sensitised relatives who come into the hospital and during re-settlement visits at their homes.

#### Inadequate food and medicine supplies

During the research period, there were inadequate food and medical supplies in the hospital, and poor nutrition and adherence to medicines interfere with patient recovery. The daughter of the elderly woman, an attendant relative at the hospital, quoted here had relapsed owing to poor adherence to medication, which meant they needed to stay in the hospital whilst she stabilised:

‘There’s a big shortage of the drugs so long as the patients are increasing daily…it is a big challenge to stay in the hospital now that the food is totally out here.’

Mental health workers also faced challenges owing to hospital-based drug shortages; as the PCO in-charge said, ‘what are we supposed to do when they bring in a violent person who is tied up with ropes? It is very dangerous not to have the drugs’. Psychiatric medicine in Uganda is lacking not only in availability but also in variety, which means that prescriptions cannot be tailored to individuals. As one former in-patient in the mental health unit said, ‘the medicine made my sickness very strange’.

Mental health workers described difficulties in promoting patients’ recovery in a challenging environment. The female senior hospital administrator at Fort Portal in her 50s, having extensive experience in Uganda’s psychiatric hospitals, said that mental healthcare requires ‘service above self’:

‘You have to have service above self. Because you work for longer hours, you are talking to a person who will not understand, you say please bathe, at the end of the day he’s getting dirty water and is making a mess. Please eat on the plate, pours food down, eats. And so, if you don’t have patience, if you don’t have that heart for them, you can’t manage. You have to be down to earth.’

## Discussion

This study shows that experiences of mental health disorders amongst respondents in the mental health unit at Fort Portal Hospital are complexly intertwined with their socio-economic context. Poverty, unemployment and financial stress were commonly recognised as both the cause and consequence of mental health problems. Stigma at institutional, community and family levels could result in further economic exclusion of people with mental health disorders, which, in turn, can lead to a worsening of the initial mental health problem. Mental health theorists in Uganda have described this relationship as a ‘vicious cycle’17 between mental health problems, poverty and stigma. Or, in other words, poverty can cause mental health problems; mental health problems can elicit stigmatisation; stigma can cause poverty and prolong mental health problems.

### Economic causes of mental health problems

Many of the mental health service users, their relatives, health workers and community leaders encountered during fieldwork in Fort Portal recognised the economy as a cause of mental health problems. Some interviewees identified the potential of unemployment to undermine mental well-being, particularly amongst the youth. This was often connected to drug and alcohol use as a coping mechanism. Financial stress was commonly recognised as a trigger for breakdown, sometimes expressed as ‘too many’ or ‘too few’ thoughts, said to be a common idiom of distress around the world.^24^ According to this idiom, excessive stress resulting from a lack of resources causes an absence of thought, with breaking down as a way of coping with contextual problems.

### Economic consequences of mental health problems

This study shows that exclusionary attitudes and behaviours towards people decreed *mulalu* may prevent access to institutions such as employment. The phrase ‘that one is mulalu’ was commonly heard during the research period, suggesting that mental health problems replace an individual’s identity. This practice is what Link and Phelan^16^ would define as ‘labelling within a power context’, identifying a person as less productive or capable of success, determining ‘life disadvantage’ and informing and reflecting inequalities at personal and institutional levels of society. In labelling and excluding people from institutions, the social meanings attached to mental health disorders can have greater longevity and damage than the health problem itself. The *mulalu* label is therefore potent and can inflict further damage on people with mental health disorders, as well as those near to them. This includes relatives and mental health workers who are discriminated by association, which disincentivises psychiatric caregiving and exacerbates systemic limitations.

### Challenges in mental healthcare

As in much of the Ugandan economy, international development aid dictates the priorities of the healthcare system,^25^ seemingly to the detriment of mental health services. This is particularly evident in relation to HIV, which, according to the 2014 estimates from the United Nations, has affected around 11.6% of the population in the Kabarole district.^26^ At Fort Portal Hospital, physical care for those with HIV/AIDS is funded by the United States Agency for International Development/Strengthening Uganda’s Systems for Treating AIDS Nationally,^27^ whilst psychological treatment for psychological breakdown, owing to organic or neurologic factors, stress or grief,^28^ is dependent on state resources. National funding limitations mean that there are sometimes inadequate food and medical supplies to treat patients at the Fort Portal mental health unit, with a detrimental effect on patient health. When the food runs out, in-patients may have to leave before they have fully recovered. Similarly, poor adherence to drugs hinders recovery and prolongs mental health problems. The absence of available drugs may leave people with mental health disorders exposed to mistreatment such as restraint, violence and incarceration.

Access to newer psychiatric medicines is restricted. For example, second-generation antipsychotics were discovered in the 1980s, and the WHO finds their side effects ‘more tolerable’ but not cost-effective in the developing world.^29^

This cost-ineffectiveness in low-income countries such as Uganda is ensured by large-scale factors such as inflated costs of non-generic pharmaceuticals and ineffective national drug procurement processes.^30^ The new medicines also come with new risks, including weight gain and diabetes, and their superiority to first-generation anti-psychotics is debated; at least 70% of comparative research studies are said to be industry funded, with bias insufficiently addressed.^30^ The introduction of these new medical risks on a large scale in Uganda is therefore not necessarily a preferable alternative, and adverse effects depend on the individual patient. However, the ‘cost-ineffectiveness’ of newer medicines in Uganda is symptomatic of a global pharmaceutical imbalance, meaning that recovery options are more limited in Uganda than elsewhere in the world. This supports quoted mental health worker’s frustrations that institutional processes hinder the accessibility of mental healthcare in Western Uganda.

## Limitations

The main limitation of this study is that there was only a single researcher, which may have introduced bias in the data analysis. However, the researcher spent 3 months in the study setting to develop an in-depth understanding of the context and experiences of the participants. Furthermore, the research assistant had long-term employment in the mental health unit of Fort Portal Hospital, as an occupational therapist. Research design was also informed by the PCO in-charge. As respondents were made up of a judgement sample, responses are not necessarily generalisable to the entire community. Judgement sampling also limits generalisability to other hospitals in Uganda and elsewhere. Further research should expand the sample of respondents, and data collection and analysis should be conducted and reviewed collaboratively.

## Conclusion

Notwithstanding the study limitations, this open-ended qualitative study provides an understanding of the socio- economic aspects of mental health within the context of Fort Portal from the perspectives of various informed respondents. The findings suggest that greater mental health awareness is needed amongst governing bodies and healthcare funders to support increased investment in services and resources, including food and medicines, outreach and sensitisation programmes, and mental health staffing and salaries. Funding in particular should target low-income and rural populations, whose socio-economic situation can hinder their mental health. Further research would assist service providers to properly respond to mental health problems and thereby counter prevailing attitudes and institutional neglect. This includes supporting existing care services, both in hospitals and within families and communities, and also designing innovative approaches to bridge gaps in mental healthcare in an efficient and cost-effective manner.

## Footnotes

i Glosbe, ‘mulalu’ translation, [No date; cited 2019 Mar 13]. Available from: https://glosbe.com/lg/en/mulalu
